# Oncologic treatment support via a dedicated mobile app: a prospective feasibility evaluation (OPTIMISE-1)

**DOI:** 10.1007/s00066-023-02166-7

**Published:** 2023-11-10

**Authors:** Fabian A. Schunn, Rami A. El Shafie, Dorothea Kronsteiner, Lukas D. Sauer, Andreas Kudak, Nina Bougatf, Dieter Oetzel, Anna Krämer, Sebastian Regnery, Timo Machmer, Jürgen Debus, Nils Henrik Nicolay

**Affiliations:** 1 Department of Radiation Oncology, Heidelberg University Hospital, Im Neuenheimer Feld 400, 69120 Heidelberg, Germany; 2 National Center for Tumor diseases (NCT), Heidelberg, Germany; 3 Clinical Cooperation Unit Radiation Oncology, German Cancer Research Center (DKFZ), Heidelberg, Germany; 4 Institute for Medical Biometry (IMBI), Im Neuenheimer Feld 130.3, 69120 Heidelberg, Germany; 5 Department of Radiation Oncology, Göttingen University Hospital, Robert-Koch-Str. 40, 37075 Göttingen, Germany; 6 Department of Radiation Oncology, University Medical Center Freiburg, Robert-Koch-Straße 3, 79106 Freiburg, Germany; 7 German Cancer Consortium (DKTK), Partner Site Freiburg, German Cancer Research Center (dkfz), Im Neuenheimer Feld 280, 69120 Heidelberg, Germany; 8 OPASCA GmbH, Mannheim, Germany

**Keywords:** Patient-reported outcome measures, Cancer, mhealth, Quality of life, Radiotherapy

## Abstract

**Background:**

Mobile health (mhealth) is gaining interest, with mobile devices and apps being ever more available among medical facilities and patients. However, in the field of radiation oncology, the medical benefits of mhealth apps are still underexplored. As an additional approach to patient care during radiotherapy, we designed a mobile treatment surveillance app based on patient-reported outcomes.

**Objective:**

We aimed to examine the feasibility of app-based treatment surveillance in patients undergoing radiotherapy (RT). Alongside technical practicability and acceptance, we assessed patient satisfaction and quality of life during treatment.

**Methods:**

This prospective single-center study was performed at Heidelberg University Hospital between August 2018 and January 2020. During RT we measured patients’ quality of life, symptoms, and treatment satisfaction. Respective questionnaires (EORTC QLQ-C30 with diagnosis-specific modules, RAND PSQ-18) were presented to patients via a mobile app running on a designated tablet device. The primary endpoint was determined by the fraction of patients who completed at least 80% of the items. Secondary endpoints were disease-related quality of life and patient satisfaction.

**Results:**

A total of 49 cancer patients (14 breast, 13 pelvic, 12 lung, 10 prostate) were eligible for analysis. 79.6% (95% confidence interval: 66.4–88.5%; *n* = 39) of all patients completed at least 80% of the items received by the mobile app. A mean of 227.5 ± 48.25 questions were answered per patient. Breast cancer patients showed the highest rate of answered questions, with 92.9% (*n* = 13) completing at least 80% of the items.

**Conclusion:**

Patients showed high acceptance, with 79.6% (*n* = 39) completing at least 80% of the given items. The use of a mobile app for reporting symptoms and quality of life during RT is feasible and well accepted by patients. It may allow for resource-efficient, detailed feedback to the medical staff and assist in the assessment of side effects over time.

**Supplementary Information:**

The online version of this article (10.1007/s00066-023-02166-7) contains supplementary material, which is available to authorized users.

## Introduction

With the rising use of mobile devices in everyday life, the use of programs or apps in the professional medical field has also increased. Specifically designed medical apps are being introduced into the landscape of treatment surveillance and follow-up [1]. The World Health Organization (WHO) summarizes medical care via the use of mobile devices under the term “mhealth” [2]. Lately, the implementation of mhealth tools in the field of oncology has been gaining interest among clinicians and researchers. A randomized prospective study compared web application-guided follow-up to regular clinical assessment in lung cancer patients [3]. App-based follow-up was found to be beneficial, resulting in significant survival improvement and better performance status at the time of relapse. It might allow for earlier relapse detection by tracking the patient’s symptoms and alerting clinicians if predefined criteria are met [4]. Recent findings of Basch et al. [5] further underline beneficial effects of web-based patient-reported outcome surveys on physical function, symptom control, and health-related quality of life.

These effects are especially desirable in oncology patient cohorts, who often undergo high-frequency treatment and follow-up regimens. A survey among German healthcare professionals revealed high willingness to implement app-based solutions in the treatment and surveillance of oncology patients [6]. However, evaluations of available medical apps in the oncologic field remain inconclusive [7]. An assessment from 2016 found most apps to be limited in function, often lacking clear information on funding and a validated scientific background [8]. Oncologic mhealth applications are mostly used for educating patients and providing pain or cancer diary functions.

More recent findings in breast cancer patients provide further support of app-based follow-up and suggest that it can assist patients in disease self-management [9]. Patients reported significantly higher disease-related quality of life when follow-up was conducted via a mobile app.

To this day, the use of mhealth concepts for oncologic treatments like radiation therapy (RT) is largely underexplored. A prospective survey conducted with patients undergoing curative RT in 2018 showed good acceptance of mobile apps for treatment surveillance and follow-up [10]. While younger patients reported being more well versed in smartphone use, overall acceptance of mobile apps in the context of RT was shown to be high across all age groups and genders. Several potential health benefits of app use by RT patients have been demonstrated in prior studies. These include improvement of health literacy, surveillance, and treatment of side effects or complications, and preservation of quality of life during aftercare [11–14]. Distinct interest lies in the development of treatment-accompaniment apps, to help guide patients undergoing therapy. Besides improving the health literacy of patients, such apps could assist radiation oncologists in reviewing symptoms under treatment, recommending supportive measures, and allocating resources accordingly. As a high-frequency treatment, typically over several weeks and with increasing symptom burden over time, RT seems to be a setting with great overall potential for mhealth applications. However, the actual implementation of these applications by healthcare providers is still lagging behind [15]. In this publication, we report the findings of a prospective feasibility study assessing app-based treatment monitoring during curative RT. We examined the overall feasibility of app-based treatment surveillance and aimed to characterize subgroups of patients according to their acceptance of this mobile approach. We also outline patient-reported quality of life and treatment satisfaction during app-accompanied RT.

## Methods

### Study design and recruitment

This prospective single-center study was performed at Heidelberg University Hospital between August 2018 and January 2020 (Trial registration: ClinicalTrials.gov NCT03168048; https://clinicaltrials.gov/ct2/show/NCT03168048 [archived at WebCite http://www.webcitation.org/6wtWGgi0X]). Patients were screened in the outpatient clinic of the Department of Radiation Oncology, University Hospital Heidelberg. Inclusion criteria were an indication for radiotherapy to the chest or pelvis in curative intent, age of 18 years or older, a good general performance score (Karnofsky performance index ≥ 70%), and a generally outpatient course of treatment. Patients who did not fulfill these criteria or who did not provide written informed consent were excluded from the study.

The goal of this study was to evaluate the feasibility and acceptance of app-based treatment surveillance of patients undergoing curative RT. To evaluate this metric, we designed a treatment-support web-application in cooperation with OPASCA GmbH Mannheim, Germany. The main component of the app was the query of items of validated EORTC (European Organisation for Research and Treatment of Cancer) and RAND (research and development) Corporation questionnaires in a predefined sequence, daily during RT. These included assessments of disease-related quality of life as well as treatment-specific symptoms and functions, patient satisfaction, and overall well-being. According to standard of care at our department, patients received a planned doctor’s consultation appointment every week. The app featured the option to request an additional consultation appointment on demand, which was generally granted on the same day. The visiting clinician had access to the patient’s app inputs to help and guide all planned and requested appointments.

The treatment-support app was run on a department-owned tablet device, which was handed to participants during the waiting period for their daily RT appointments. During this time, they were asked to complete the items presented on that day and were offered to request a consultation appointment. The app design was very intuitive and easy to use, and no special patient education was required (screenshot example displayed in supplementary Multimedia Appendix 1). If required, radiation therapist personnel provided assistance. Participants spent around 5–10 min per day with the app and handed the tablet device back to the RT technician at the initiation of each RT session. This approach with a central input device was necessary for this pilot study due to infrastructural and data security reasons, as well as local policies. Future iterations of the app, however, will run on the participants’ personal mobile devices.

Treatment duration, dose prescription, and technical aspects of RT followed the general standard of care at the radiation oncology department of Heidelberg University Hospital. RT was performed once daily, with five fractions per week (Monday through Friday). Participation in this study did not affect the planning or course of RT. Treatment was performed in an outpatient setting, with the exception of patients receiving concomitant chemotherapy. In those cases, patients could be admitted to a hospital ward for typically 1–2 days per dose of chemotherapy, if necessary.

### Data collection

**Fig. 1:**
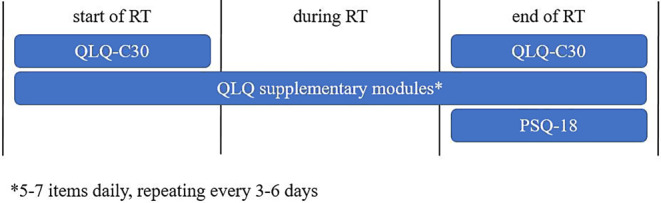
Study visits and corresponding questionnaires; the number of visits during *RT* varied due to length of treatment and instruments used

Within the treatment-support app, patients were asked to complete the EORTC QLQ-C30 questionnaire on the first and last day of RT, as well as the RAND PSQ-18 questionnaire on the last day of RT. Five to seven items of supplementary diagnosis-specific EORTC QLQ modules were presented daily during RT, repeating after 3–6 days (Fig. 1). The supplementary modules used were BR23 for breast cancer; CX24 for vaginal, cervical, or endometrial cancer; CR29 for rectal cancer; LC13 for lung cancer; and PR25 for prostate cancer patients. In summary, patients received between 115 and 291 app-based questions over the course of RT, depending on treatment duration and the modules used. The primary exploratory endpoint of feasibility was defined by the fraction of patients who completed at least 80% of all app-based questions. Secondary endpoints were disease-related quality of life and patient satisfaction. Also, patients were able to request a doctor’s consultation via the app, resulting in more thorough medical care compared to established treatment standards. A sample screenshot of the app surface is provided in the electronic supplementary materials (Multimedia Appendix 1). In the context of patient screening, we inquired about the use of mobile devices for personal and medical purposes using a self-designed smartphone questionnaire. These screening items were paper based and did not count towards the primary endpoint.

All data collected within this study was pseudonymized and stored on a central department-owned on-site server to which only the study conductors had access. The only exception to this was in the case of a requested doctor’s consultation appointment, where all app-based inputs where automatically forwarded to the visiting clinician for guidance. After study completion, a database extract of the study data was archived in the clinic’s clinical study archive and deleted from the server, which is being used for further development.

### Instruments

Patient-reported quality of life was evaluated with the EORTC QLQ-C30 questionnaire and its supplementary modules. The QLQ-C30 is a widely used tool for the measurement of quality of life in cancer patients provided by the European Organization for Research and Treatment of Cancer (EORTC). It consists of 30 items assessing five functional (physical, role, emotional, cognitive, social) and nine symptomatic (fatigue, nausea and vomiting, pain, dyspnea, insomnia, appetite loss, constipation, diarrhea, financial difficulties) aspects of health-related quality of life in 15 subscales, including a global health status scale. Higher scores on a functional scale express better respective functioning, while higher symptom scale scores indicate a higher symptomatic burden. Overall, the QLQ-C30 has been found to be highly reliable and consistent in the measurement of quality of life and is well established in cancer research [16]. While it provides a valid general evaluation of quality of life, several supplementary modules have been established for the assessment of more diagnosis- and treatment-specific issues patients might encounter. Generally, the supplementary modules follow the same scoring procedures as the QLQ-C30, providing additional disease-specific functional and symptom scales [17, 18]. For example, the breast cancer module BR23 contains items on body image and sexual functioning, while the lung cancer module LC13 queries typical symptoms like coughing, hemoptysis, and chest pain. Use of the QLQ-C30 within a mobile app has been found to be well accepted by patients [19].

The patient satisfaction questionnaire short form (PSQ-18) was used to assess patient satisfaction at the end of RT. It is a patient-reported measurement tool for satisfaction with medical care, provided by the RAND (research and development) Corporation as an 18-item short form of the 50-item PSQ-III [20]. Items are scored on a five-point Likert scale and can be grouped into seven dimensions of treatment satisfaction (general satisfaction, technical quality, interpersonal manner, communication, financial aspects, times spent with doctor, and accessibility and convenience). Response values are converted to score values, so that a higher score represents greater satisfaction. The PSQ-18 has been proven to be internally consistent and reliable, and is substantially correlated with its corresponding long form.

### Ethical aspects

All work in relation to this study followed the Declaration of Helsinki [21] and was approved by the institutional ethics committee. Patient involvement was voluntary, and no disadvantages resulted from declining participation. Informed written consent was obtained prior to enrollment in the study. Participants were provided detailed information on the collection and storage of data, as well as the option to withdraw consent at any time during the investigation. All personal information of participants was pseudonymized after data collection.

### Statistical analysis

Descriptive statistical analysis of EORTC questionnaires was performed using R version 4.0.2 and the supplemental packages QoLR and PROscore (R: A language and environment for statistical computing. R Foundation for Statistical Computing, Vienna, Austria; URL https://www.R-project.org/). For the QLQ-C30 and its supplementary modules, a linear transformation of raw scores was performed to achieve values between 0 and 100, in accordance with the EORTC manual. Missing items were imputed by mean of the other scale items if clinically reasonable and if at least half of the items had been answered. Patients who answered fewer items were omitted for that timepoint.

In subgroup analysis, chi-squared test and *t*-test were preformed to test for significant differences between the groups. A *p*-value of < 0.05 was deemed statistically significant. Wilcoxon’s one-sample signed-rank test for paired data was used for the evaluation of the QLQ-C30 questionnaire.

## Results

### Patient characteristics

A total of 54 patients was recruited, including four screening failures (three due to changes in treatment regimen, one due to withdrawal of consent) and one dropout due to hospitalization, leaving 49 patients eligible for analysis. The final study population was made up of 28.6% (*n* = 14) breast cancer, 24.5% (*n* = 12) lung cancer, 22.5% (*n* = 11) vaginal/cervical/endometrial cancer, 20.4% (*n* = 10) prostate cancer, and 4.1% (*n* = 2) rectal cancer patients. Vaginal, cervical, endometrial, and rectal cancer patients were pooled under “pelvic cancer” for further data analysis. Sociodemographic and clinical features of patients are shown in Table 1. Mean age was 59.0 ± 11.9 years at the beginning of treatment. The study population consisted of 59.2% (*n* = 29) female and 40.8% (*n* = 20) male participants. A majority of 77.6% of patients (*n* = 38) stated owning a smartphone for regular personal use.

**Table 1:** Patient characteristics and demographics

	Total (*n* = 49)
*Age (years)*	
Mean	59
Standard deviation	11.94
Median	60
Range	29–79
*Sex*	
Female	29 (59.2%)
Male	20 (40.8%)
*Regular smartphone use*	
Yes	38 (77.6%)
No	8 (16.3%)
*Tumor diagnosis*	
Breast	14 (28.6%)
Pelvic	13 (26.5%)
Lung	12 (24.5%)
Prostate	10 (20.4%)

### Primary endpoints

Results for descriptive analysis of the exploratory primary endpoint of feasibility are shown in Table 2. Depending on diagnosis and treatment procedure, patients received between 15 and 35 fractions of radiotherapy, with a median of 28 fractions. One fraction was applied on every workday, with five fractions per week. During this time, patients were asked to complete 243.6 ± 48.25 items on average, ranging from 115 to 291 items. Over all subgroups, 79.6% (95% confidence interval [CI]: 66.4–88.5%; *n* = 39) of patients completed at least 80% of all app-based items. A mean of 227.5 ± 48.25 items was completed per patient. Breast cancer patients showed the highest rate of completion, with 92.9% (*n* = 13) completing at least 80% of the items. The lowest rate of completion was observed among pelvic cancer patients, with 61.5% (*n* = 8) completing 80% of the items.

**Table 2:** RT fractions, total number of items presented by the app, amount of completed items, fraction of at least 80% completion; grouped by tumor diagnosis and in total

	Breast cancer (*n* = 14)	Pelvic cancer (*n* = 13)	Lung cancer (*n* = 12)	Prostate cancer (*n* = 10)	Total (*n* = 49)
*RT fractions*					
Mean	21.4	27.2	31.2	34.2	28
SD	4.57	2.08	1.54	0.42	5.55
Median	25	28	30	34	28
Range	15–25	25–28	30–33	34–35	15–35
*Total items*					
Mean	204.4	239.6	278.9	286.2	248.7
SD	31.04	12.74	9.78	2.53	37.58
Median	221	244	271	285	249
Range	115–221	226–275	271–290	285–291	115–291
*Completed items*					
Mean	197.9	207.1	256.8	260.2	227.5
SD	38.96	43.90	44.24	31.00	48.25
Median	219	209	268.5	270	221
Range	115–221	118–275	147–290	206–291	115–291
*80% completion*					
Yes	13 (92.9%)	8 (61.5%)	10 (83.3%)	8 (80%)	39 (79.6%)
No	1 (7.1%)	5 (38.5%)	2 (16.7%)	2 (20%)	10 (20.4%)

*SD* standard deviation

We performed a subgroup analysis for the variables tumor diagnosis, age, sex, smartphone use, and quality of life at the beginning of RT (global health scale QL of the EORTC QLQ-30 questionnaire) to test for potential effects on the 80% completion rate. Results are reported in Table 3. Age was the only variable found to be significantly different between the groups. Patients with a completion rate ≥ 80% were on average 8.8 years younger than those with a completion rate of < 80%. For tumor diagnosis, sex, regular smartphone use, and quality of life, no significant differences could be found.

**Table 3:** Subgroup analysis of tumor diagnosis, age, sex, smartphone use, and quality of life at the beginning of RT in relation the completion rate of items

	Completion < 80% (*n* = 10)	Completion ≥ 80% (*n* = 39)	Total (*n* = 49)	*p*-value
*Tumor diagnosis*				
Breast	1 (10.0%)	13 (33.3%)	14 (28.6%)	0.582
Pelvic	5 (50.0%)	8 (20.5%)	13 (26.5%)	
Lung	2 (20.0%)	10 (25.6%)	12 (24.5%)	
Prostate	2 (20.0%)	8 (20.5%)	10 (20.4%)	
*Age*				
Mean	66.0	57.2	59	0.036
SD	8.73	12.07	11.94	
Median	65	57	60	
Range	50–79	29–78	29–79	
*Sex*				
Female	5 (50.0%)	24 (61.5%)	29 (59.2%)	0.508
Male	5 (50.0%)	15 (38.5%)	20 (40.8%)	
*Regular smartphone use*				
Yes	9 (90.0%)	29 (80.6%)	38 (82.6%)	0.486
No	10 (10.0%)	7 (19.4%)	8 (17.4%)	
*Quality of life*				
Mean	41.7	36.4	36.9	0.467
SD	22.57	12.62	13.54	
Median	50	33.3	33.3	
Range	8.3–58.3	16.7–58.3	8.3–58.3	

*SD* standard deviation

### Secondary endpoints

Quality of life was assessed with the EORTC QLQ-C30 at the start and the end of RT, results are reported in Table 4 (functional scales) and Table 5 (symptom scales). No significant decline in global health (QL) status was observed between visits. Physical functioning (PF) and cognitive functioning (CF) showed a significant decline between visits among functional scales. Among symptom scales, fatigue (FA), and diarrhea (DI) were increased at the end of RT. Other subscales did not show significant changes between beginning and end of RT.

**Table 4:** Comparison of QLQ-C30 functional scales between beginning and end of RT

Functional scale	Visit 1 (*n* = 49)	Visit 2 (*n* = 47)	*p*-value	Functional scale	Visit 1 (*n* = 49)	Visit 2 (*n* = 47)	*p*-value
*QL*				*EF*			
Mean	37	35	0.389	Mean	55	52	0.173
SD	14	12		SD	27	26	
Median	33	33		Median	58	50	
Range	8.3–58	8.3–67		Range	8.3–100	0–92	
*PF*				*CF*			
Mean	80	73	0.041	Mean	83	76	0.039
SD	19	26		SD	19	25	
Median	87	80		Median	83	83	
Range	33–100	6.7–100		Range	33–100	0–100	
*RF*				*SF*			
Mean	53	55	0.904	Mean	66	60	0.096
SD	34	35		SD	31	33	
Median	50	50		Median	67	67	
Range	0–100	0–100		Range	0–100	0–100	

*QL* quality of life, *PF* physical functioning, *RF* role functioning, *EF* emotional functioning, *CF* cognitive functioning, *SF* social functioning, *SD* standard deviation

**Table 5:** Comparison of QLQ-C30 symptom scales between beginning and end of RT

Symptom scale	Visit 1 (*n* = 49)	Visit 2 (*n* = 47)	*p*-value	Symptom scale	Visit 1 (*n* = 49)	Visit 2 (*n* = 47)	*p*-value
*FA*				*AP*			
Mean	44	55	0.024	Mean	17	24	0.052
SD	30	31		SD	25	32	
Median	39	56		Median	0	0	
Range	0–100	0–100		Range	0–100	0–100	
*NV*				*CO*			
Mean	6.3	13	0.059	Mean	25	19	0.121
SD	14	20		SD	32	27	
Median	0	0		Median	0	0	
Range	0–67	0–83		Range	0–100	0–100	
*PA*				*DI*			
Mean	33	36	0.479	Mean	8.7	23	0.013
SD	32	27		SD	18	31	
Median	33	33		Median	0	0	
Range	0–100	0–100		Range	0–67	0–100	
*DY*				*FI*			
Mean	29	35	0.467	Mean	14	20	0.053
SD	36	34		SD	25	29	
Median	0	33		Median	0	0	
Range	0–100	0–100		Range	0–100	0–100	
*SL*				–			
Mean	45	50	0.395				
SD	34	33					
Median	33	33					
Range	0–100	0–100					

*FA* fatigue, *NV* nausea and vomiting, *PA* pain, *DY* dyspnea, *SL* insomnia, *AP* appetite loss, *CO* constipation, *DI* diarrhea, *FI* financial difficulties, *SD* standard deviation

**Fig. 2:**
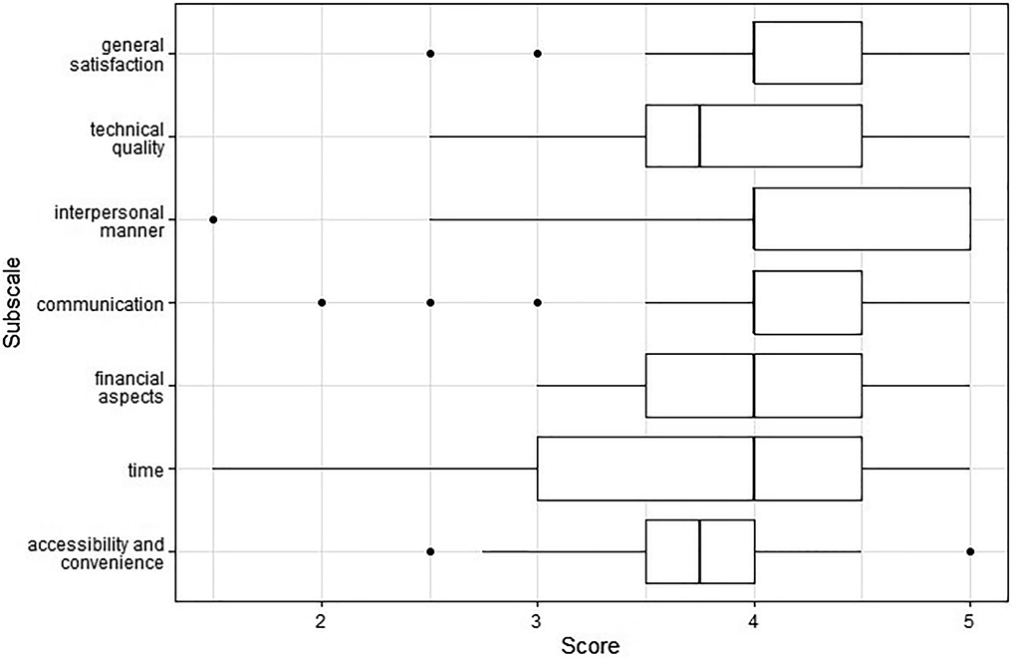
PSQ-18 score values by subscales

Patient satisfaction was reported at the end of RT by means of the PSQ-18 questionnaire. Overall, patient satisfaction was high across all subscales. Score values of PSQ-18 subscales are depicted in Fig. 2.

#### Supplementary QLQ modules

**Fig. 3:**
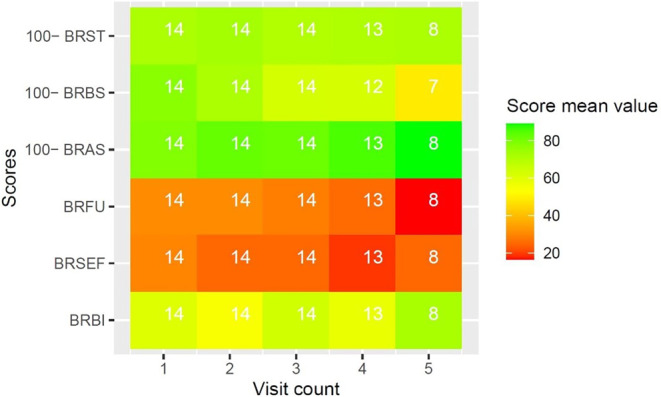
Heatmap of BR23 score mean values for systemic side effects (*BRST*), breast symptoms (*BRBS*), arm symptoms (*BRAS*), future perspective (*BRFU*), sexual functioning (*BRSEF*), and body image (*BRBI*) over five visits

**Fig. 4:**
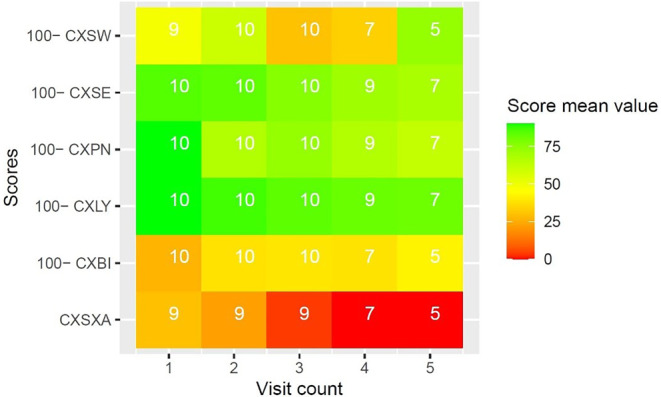
Heatmap of CX24 score mean values for sexual worry (*CXSW*), sexual enjoyment (*CXSE*), peripheral neuropathy (*CXPN*), lymphoedema (*CXLY*), body image (*CXBI*), and sexual activity (*CXSXA*) over five visits

**Fig. 5:**
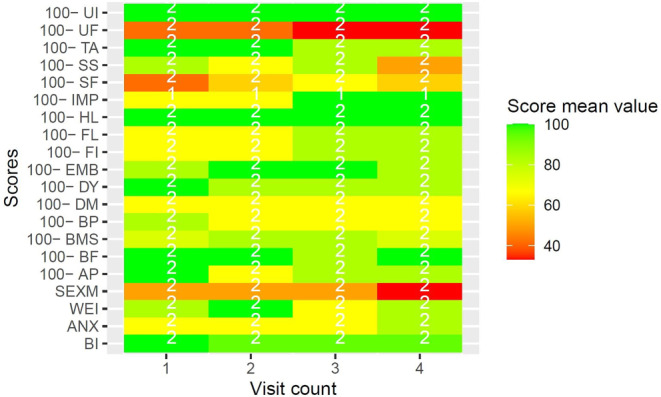
Heatmap of CR29 score mean values for urinary incontinence (*UI*), urinary frequency (*UF*), trouble with taste (*TA*), sore skin (*SS*), stool frequency (*SF*), impotence (*IMP*), hair loss (*HL*), flatulence (*FL*), fecal incontinence (*FI*), embarrassed by bowel movement (*EMB*), dysuria (*DY*), dry mouth (*DM*), buttock pain (*BP*), blood and mucus in stool (*BMS*), bloated feeling (*BF*), abdominal pain (*AP*), sexual function men (*SEXM*), weight (*WEI*), anxiety (*ANX*), and body image (*BI*) over four visits

**Fig. 6:**
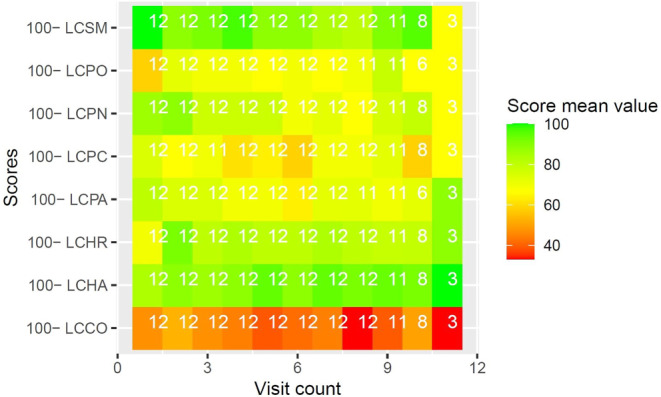
Heatmap of score mean values for sore mouth (*LCSM*), pain in other parts (*LCPO*), peripheral neuropathy (*LCPN*), pain in chest (*LCPC*), pain in arm or shoulder (*LCPA*), alopecia (*LCHR*), hemoptysis (*LCHA*), and coughing (*LCCO*) over 11 visits

**Fig. 7:**
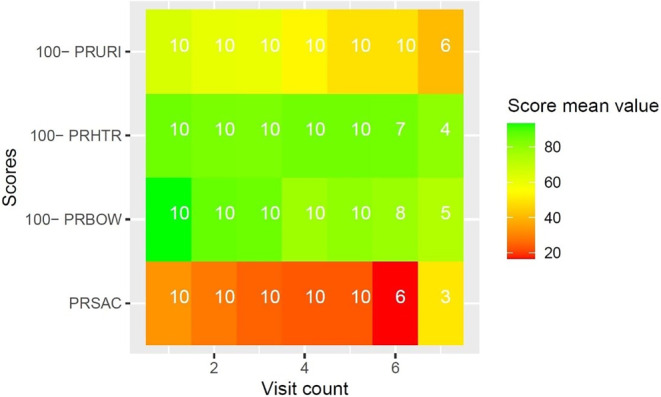
Heatmap of score mean values for urinary symptoms (*PRURI*), hormonal treatment (*PRHTR*), bowel symptoms (*PRBOW*), and sexual activity (*PRSAC*) over seven visits

Diagnosis-specific quality of life under RT was evaluated with EORTC QLQ supplementary modules. Due to the high number of datapoints, and to preserve clearness and brevity, results of EORTC QLQ supplementary modules are reported visually in Figs. 3, 4, 5, 6 and 7. Heatmaps were used to visualize score mean values, case numbers, and visit counts of EORTC QLQ supplementary modules. Score mean values are depicted by tile coloring, with green coloring generally representing a medically favorable result. To achieve a uniform representation, score mean values of symptom scales were inverted (subtracted from 100), thus matching the coloring scheme for functional scales. Numbers inside the tile grid indicate the respective case number (*n*) of the corresponding visit count and scale. Some scales were omitted from visual representation due to missing items, low case numbers or non-applicability during RT, namely BRHL, BRSEE, CR-IMP, CR-SEXW, CR-STO, CR-DYS, CX-SXE, CX-SV, LC-DS, LC-DY, PR-AID, PR-SFU^1^. Differences in the number of items per questionnaire and overall duration of RT led to varying visit counts among supplementary modules. Visit counts ranged from 4 (CR29) to 12 (LC13). The datasets for this study are available from the authors upon reasonable request.

## Discussion

### Study findings

An app-based approach for treatment surveillance under RT proved feasible and was well accepted by patients. Overall, patients showed high participation and compliance, with 79.6% completing at least 80% of items presented. This result confirms the expectations of a 2018 survey conducted at the same institution, where 73.3% of patients expressed interest in using a mobile app for support under RT [10]. Similar rates of acceptance were also observed in earlier studies [22–24]. In a study using the “PROMetheus” app for example, 81% of patients regularly submitted data during and after RT [22]. However, investigator criteria for a good acceptance and frequent use may vary between publications. A selection of participation rates of comparable studies can be found in Table 6.

**Table 6:** Participation and adherence rates of comparable studies

Investigator	App used	Participation rate	Comment
Present study: Schunn et al. (2023)	OPTIMISE	79.6%^a^ (95%CI: 66.4–88.5%)	Defined as completing at least 80% of all presented items
Hauth et al. (2019) [22]	PROMetheus	81%^a^	Defined as at least weekly reports
Langius-Eklöf et al. (2017) [23]	Interaktor	87%^a^ (range 16–100%)	Defined as adherence to daily reporting
Crafoord et al. (2021) [24]	Interaktor	83%^b^ (IQR 36%)	Defined as adherence to daily reporting

*CI* confidence interval, *IQR* interquartile range

^a^Mean value

^b^Median value

Age was the only variable found to differ significantly between patients with an item completion rate over or under the 80% cutoff. Younger patients seemed to be more compliant with the use of app-based treatment surveillance. In our collective, breast cancer patients showed the best compliance and were also the youngest group, with a mean age of 51.6 ± 9.3 years. These finding are in line with a 2017 study on app-assisted cancer care that also found younger patients to be more accepting of app-based care [25]. While regular smartphone use was reported by a majority of patients (77.6%), this variable did not have a significant effect on item completion rate. This indicates that even patients with less smartphone proficiency may find an app-based treatment surveillance accessible. In the same manner, a lower quality of life did not seem to impact the completion rate in a negative way. Overall, app-based treatment surveillance as conducted in this study seems to pose a low compliance threshold for patients during curative RT. Similar results were produced by a 2021 study on young cancer patients that found app use to be unaffected by sociodemographic factors and tumor stage [26].

Regarding the secondary endpoints, several trends can be observed in the results of EORTC questionnaires. Patients generally reported lower disease-related quality of life in EORTC QLQ supplementary modules towards the end of RT. This indicates higher symptomatic burden and lower functionality at the end of treatment, which can be explained in part by increasing RT side effects. Comparison of QLQ-C30 subscales showed an increased occurrence of fatigue (FA) and diarrhea (DI) at the end of treatment, both of which are common RT side effects. Loss of appetite (AP) and nausea (NV) were also more common at the end of treatment, though *p*-values were slightly above the significance threshold. With regard to the small sample size, this can still be considered a meaningful effect. Global health status (QL), however, did not decline significantly between visits. We conclude that the daily query of symptoms allows for detection of minute changes in quality of life, even if RT is generally well tolerated.

Case number decreased with visit count for most EORTC QLQ scales. This is partly due to differences in the duration of RT, with patients in shorter treatment regimens not participating in the final visit count. However, it might also indicate decreasing compliance over time, which has already been demonstrated in prior mhealth trials among cancer patients [27]. While we could not quantify this metric in detail, we find it important to point out the effects that RT itself might have on patients’ quality of life and compliance over time. Additionally, decreasing compliance might be an expression of patients feeling overburdened with questions, and has to be considered thoroughly during app development and in future projects.

Patient satisfaction at the end of treatment was evaluated with the RAND PSQ-18 questionnaire. Overall, patient satisfaction was high when compared to normative values [20]. Besides outliers, no significant deficits were found in the different aspects of patient satisfaction. These findings underline the high acceptance of app-based treatment surveillance as performed in this study.

According to the findings of our study, treatment surveillance with the developed app seems to be feasible and possibly contribute to patient satisfaction with the performed treatment. This is a promising prospect for further implementation of this approach in our clinic, as well as continued development of the evaluated app. Central feature upgrades planned for future versions of the app include the following: deployment on the patient’s own mobile device including email and push notifications for survey completion. This includes the need for safe data communication through the clinic firewall and data pseudonymization outside of clinic systems to adhere to EU data protection regulations. Furthermore, additional functionality including appointment booking and reminders are planned, as widely requested in a large multicenter survey conducted among radiotherapy patients [10]. To achieve better integration of the app into the clinical workflow, as well as transferability to other clinical ecosystems, the development of a communications interface based on current Health Level 7 (HL‑7) and Fast Healthcare Interoperability Resources (FHIR) protocols is planned. Following implementation of these features, a randomized trial is warranted to quantify the objective benefit achieved for oncology patients.

### Strengths and limitations

The prospective and rigorous approach and broad patient spectrum of this study are notable advantages in the assessment of feasibility of app-based treatment support. By using well-established questionnaires, we accounted for the need for scientifically validated tools in the field of mhealth.

Several limitations of this study must be considered. Firstly, the number of cases was limited to 49 participants. While this allows for sound statistical analysis of the overall study population within the scope of a feasibility study, it limits the possibilities of subgroup evaluation. Secondly, patients were aware of their participation in a clinical study. This might result in socially desirable behavior and lead them to process more items than they would have in a routine RT setting. Also, the use of a centralized investigator-owned input device might result in a higher rate of completed items. For future investigations, apps running on the patient’s personal smartphone would be preferable, as this would represent a fully integrated mhealth approach more genuinely.

## Conclusion and implications

Our findings strongly support the use of app-based treatment support for patients during curative RT. Established and versatile quality of life measurement tools like the EORTC questionnaires can be used to evaluate treatment side effects and overall well-being efficiently. In future investigations, the performance of randomized controlled trials to test for supposed beneficial effects of app-based treatment surveillance would be desirable. Also, the implementation of apps into clinical workflows and the resulting benefits for radiation oncologists need to be explored further. Wider patient cohorts including palliative care patients, and broader app functionality such as language support and accessibility appear necessary to unlock the full potential of mhealth in radiation oncology.

### Supplementary Information

Multimedia Appendix 1: sample screenshot of the OPASCA app surface

## Appendix

**Table 7:** Abbreviations for EORTC QLQ modules and subscales

**QLQ-BR23 (breast cancer module)**	
BRAS	arm symptoms
BRBI	body image
BRBS	breast symptoms
BRFU	future perspective
BRHL	upset by hair loss
BRSEE	sexual enjoyment
BRSEF	sexual functioning
BRST	systemic side effects
**QLQ-C30 (quality of life)**	
A	appetite loss
CF	cognitive functioning
CO	constipation
DI	diarrhea
DY	dyspnea
EF	emotional functioning
FA	fatigue
FI	financial difficulties
NV	nausea and vomiting
PA	pain
PF	physical functioning
QL	quality of life
RF	role functioning
SF	social functioning
SL	insomnia
**QLQ-CR29 (colorectal cancer module)**	
ANX	anxiety
AP	abdominal pain
BF	bloated feeling
BI	body image
BMS	blood and mucus in stool
BP	buttock pain
DM	dry mouth
DY	dysuria
DYS	dyspareunia
EMB	embarrassed by bowel movement
FI	fecal incontinence
FL	flatulence
HL	hair loss
IMP	impotence
SEXM	sexual function men
SEXW	sexual function women
SF	stool frequency
SS	sore skin
STO	stoma care problems
TA	trouble with taste
UF	urinary frequency
UI	urinary incontinence
WEI	weight
**QLQ-CX24 (cervical cancer module)**	
CXBI	body image
CXLY	lymphoedema
CXPN	peripheral neuropathy
CXSE	sexual enjoyment
CXSV	sexual/vaginal functioning
CXSW	sexual worry
CXSXA	sexual activity
CXSXE	sexual enjoyment
**QLQ-LC13 (lung cancer module)**	
LCCO	coughing
LCDS	dysphagia
LCDY	dyspnea
LCHA	hemoptysis
LCHR	alopecia
LCPA	pain in arm or shoulder
LCPC	pain in chest
LCPN	peripheral neuropathy
LCPO	pain in other parts
LCSM	sore mouth
**QLQ-PR25 (prostate cancer module)**	
PRAID	incontinence aid
PRBOW	bowel symptoms
PRHTR	hormonal treatment
PRSAC	sexual activity
PRSFU	sexual functioning
PRURI	urinary symptoms

## Abbreviations

EORTC: European Organisation for Research and Treatment of Cancer
mhealth: Mobile health
PSQ-18: Patient satisfaction questionnaire short form
QLQ: Quality of life questionnaire
RAND: Research and Development Corporation
RT: Radiotherapy
SD: Standard deviation
WHO: World Health Organization

## Footnotes

1 Breast cancer module (BR): HL = upset by hair loss, SEE = sexual enjoyment; colorectal cancer module (CR): IMP = impotence, SEXW = sexual function women, STO = stoma care problems, DYS = dyspareunia; cervical cancer module (CX): SXE = sexual enjoyment, SV = sexual/vaginal functioning; lung cancer module (LC): DS = dysphagia, DY = dyspnea; prostate cancer module (PR): AID = incontinence aid, SFU = sexual functioning.
