# Supplementary material for: Oncologic treatment support via a dedicated mobile app: a prospective feasibility evaluation (OPTIMISE-1)
Source: Strahlenther Onkol. 2023 Nov 10;200(6):475–86. doi: 10.1007/s00066-023-02166-7 (PMC11111550; doi:10.1007/s00066-023-02166-7)
Supplement: Supplementary file 1 — Multimedia Appendix 1: sample screenshot of the OPASCA app surface [file 66_2023_2166_MOESM1_ESM.docx]

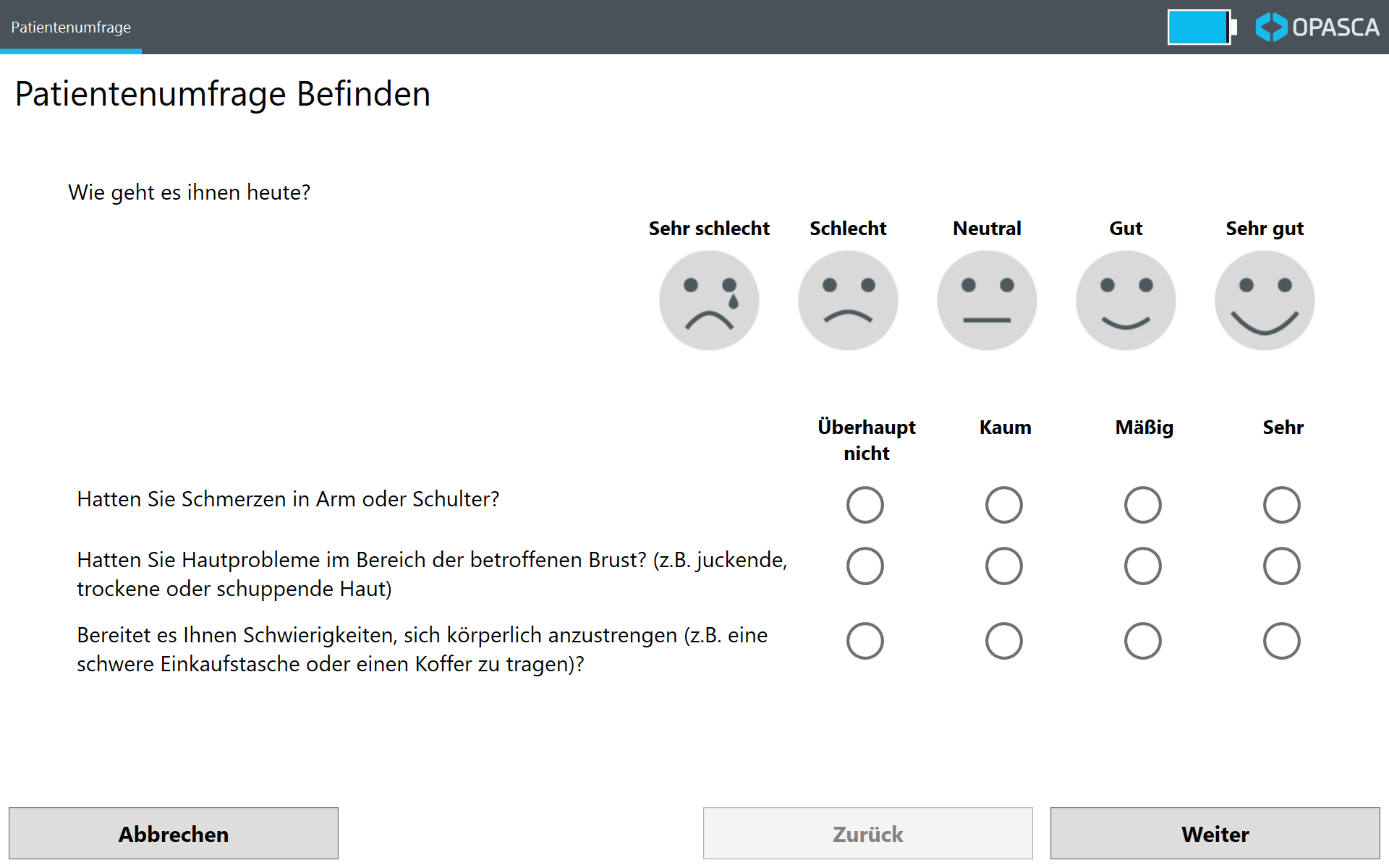


*Multimedia Appendix 1: sample screenshot of the OPASCA app surface^1^*

^1^Translation: How are you feeling today? – very bad, bad, neutral, good, very good
Have you had any pain in your arm or shoulder? / Have you had skin problems on or in the area of your affected breast (e.g., itchy, dry, flaky)? / Have you had problems with bodily exertion (e.g., carrying a heavy shopping bag or suitcase)? – not at all, a little, quite a bit, very much
